# Cutaneous dual nontuberculous mycobacterial infection in an immunocompetent adolescent male

**DOI:** 10.1016/j.jdcr.2026.01.021

**Published:** 2026-01-29

**Authors:** Connie Cai, Michelle A. Robinson, Jeffrey A. Sanford, Robert J. Smith

**Affiliations:** a Department of Dermatology, Johns Hopkins School of Medicine, Baltimore, Maryland; b Department of Dermatology, Emory University School of Medicine, Atlanta, Georgia

**Keywords:** cutaneous nontuberculous mycobacterial infection, nontuberculous mycobacterium, *Mycobacterium chelonae*, *Mycobacterium septicum*, mixed NTM infection

## Introduction

Cutaneous infection from mixed nontuberculous mycobacteria (NTM) species is rare.^1^ To date, no documented cases of a dual *M. chelonae* and *M. septicum* cutaneous infections have been reported in the literature. We present a case of NTM cutaneous infection in an immunocompetent individual in which both *M. chelonae* and *M. septicum* were isolated from tissue cultures.

## Case report

A 15-year-old immunocompetent male patient with a history of moderately severe atopic dermatitis presented to the pediatric dermatology clinic with a 6-month history of a worsening, mildly pruritic eruption on the dorsal hands. Due to a history of hand-predominant dyshidrotic eczema, he had been applying mid- to high-potency topical corticosteroids to the affected areas regularly over the preceding months.

**Fig 1:**
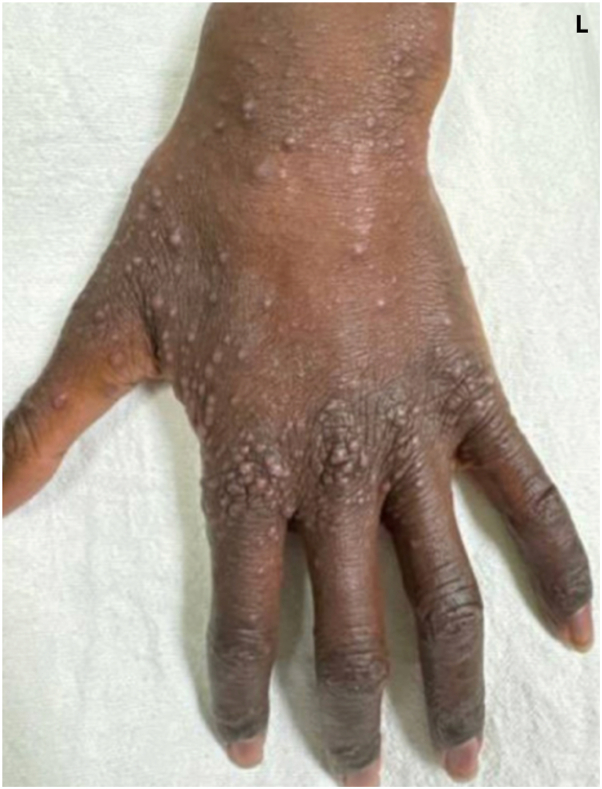
Left dorsal hand demonstrating diffuse monomorphic papules at initial evaluation.

**Fig 2:**
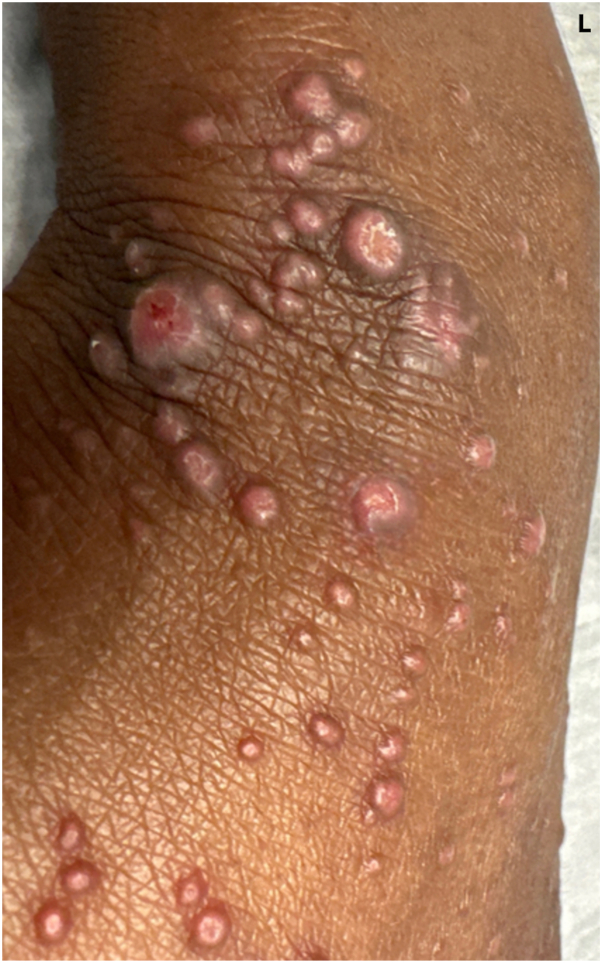
Closer image of papules on the left dorsal hand.

On physical examination, there were diffuse monomorphic pink-to-violaceous dome-shaped, flat-topped, umbilicated papules of the dorsal fingers, hands, and wrists, with notable sparing of the palms (Figs 1 and 2).

**Fig 3:**
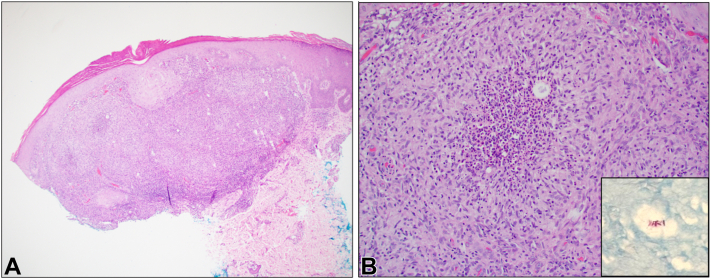
**A,** Punch biopsy specimen, H&E, original magnification 40×. Low-power photomicrograph showing a vaguely nodular, granulomatous infiltrate within the dermis. **B,** Punch biopsy specimen, H&E, original magnification 200×. High-power photomicrograph demonstrating suppurative epithelioid granulomas with multinucleated giant cells surrounded by a mixed lymphocytic and neutrophilic infiltrate. Acid-fast bacilli are present within areas of suppuration (inset, Ziehl-Neelsen stain, original magnification 400×).

A punch biopsy performed on a representative papule on the right dorsal hand demonstrated a granulomatous dermatitis concerning for infection (Fig 3, *A* and *B*). Tissue culture grew both *M. chelonae* and *M. septicum*.

With guidance from colleagues in infectious disease, he was empirically treated with doxycycline (100 mg twice daily) and azithromycin (500 mg daily). Further history revealed that he frequently washed dishes at home with rubber gloves that were later found to be contaminated with black mold. This contamination was recognized only upon follow-up, after the cutaneous diagnosis and treatment course had already been established.

**Fig 4:**
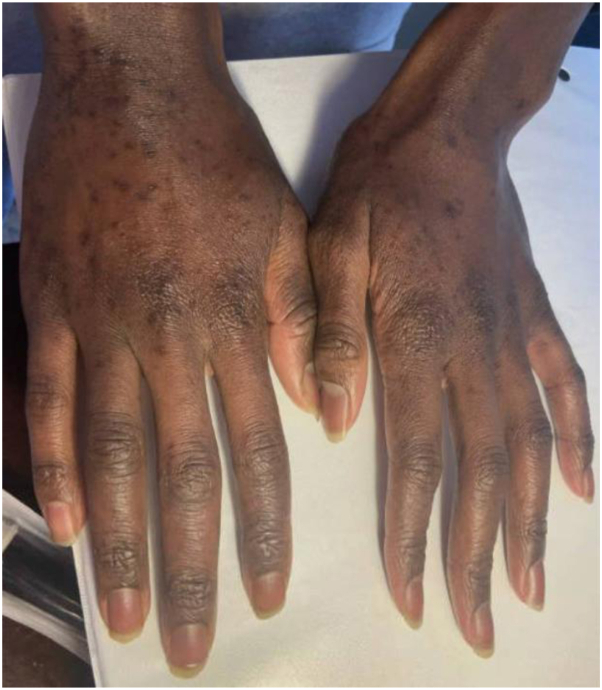
Physical examination on follow-up. Exam demonstrated scattered hyperpigmented macules at the sites of prior monomorphic papules.

After susceptibilities resulted, he was transitioned to trimethoprim/sulfamethoxazole (1600-320 mg 3 times daily) and maintained on azithromycin (500 mg daily) for a total of 5 months of therapy. Due to the unusual presence of both NTM species and the multifocal nature of his eruption, systemic immunodeficiency was considered but ruled out after immunology referral. On clinical follow-up, he was noted to have post-inflammatory hyperpigmentation with no active lesions, and antibiotic therapy was discontinued (Fig 4).

## Discussion

Our report describes a rare dual NTM infection in an immunocompetent adolescent male. Despite being immunocompetent, the patient had several potential risk factors that may have increased susceptibility to NTM infection, including a disrupted skin barrier from chronic atopic dermatitis, a blunted local immune response due to prolonged use of high-potency topical corticosteroids, and repeated exposure to moist, contaminated dishwashing gloves. Although cultures of the contaminated gloves were not performed, the visible black mold was suspected to represent an environmental nidus that may have facilitated inoculation with nontuberculous mycobacteria.

Clinically, *M. chelonae* infections present as erythematous papules, nodules, or pustules that may ulcerate or form abscesses. Lesions often persist despite standard antibiotics, prompting further microbiologic evaluation.^2^ In severely immunocompromised individuals, such as those on chronic corticosteroid therapy or immunosuppressive regimens, *M. chelonae* can cause disseminated disease.^2^

*M. septicum* isolates have uncertain clinical significance, and it is often not considered a true pathogen. It is primarily associated with catheter-related infections, and only a few cases of *M. septicum* cutaneous infections have been documented.^3^^,^^4^^,^^5^ Two previously documented cases involved immunocompetent individuals with significant physical trauma from motor vehicle accidents resulting in multiple open fractures and subsequent surgical interventions, and it was thought that *M. septicum* grown from tissue cultures in these cases represented a contaminant.^3^ Another case involved an immunocompetent individual who developed persistent eyelid masses following a blepharoplasty and was found to have *M. septicum* infection through polymerase chain reaction of tissue samples taken from the surgical site.^5^ The role of M. septicum in our patient remains uncertain, but co-isolation with *M. chelonae* suggests it may have contributed to the clinical presentation.

Treatment of *M. chelonae* typically involves dual therapy with a macrolide (eg, clarithromycin or azithromycin) and amikacin for invasive or refractory cases, while localized infections may respond to clarithromycin monotherapy.^2^ However, empiric monotherapy for atypical mycobacteria is generally contraindicated due to the risk of resistance. *M. chelonae* is resistant to cefoxitin, whereas *M. septicum*, in contrast, is resistant to clarithromycin and doxycycline but remains susceptible to amikacin, ciprofloxacin, imipenem, linezolid, moxifloxacin, and trimethoprim-sulfamethoxazole. Due to its rarity, there are no established treatment guidelines for *M. septicum*.^3^

The rarity of *M. septicum* infections likely explains the absence of previously reported dual *M. chelonae* and *M. septicum* cutaneous infections.^6^ Given that most *M. septicum* isolates have been incidental, its presence here may represent an environmental contamination or an underrecognized pathogenic potential. In contrast, *M. chelonae* is a well-established cause of cutaneous infections, making it the more likely driver of this patient’s disease presentation.

In conclusion, we report the first documented case of mixed *M. chelonae* and *M. septicum* cutaneous infection. Key risk factors included chronic atopic dermatitis, topical steroid use, and repeated environmental exposure. Limitations include the absence of glove cultures, though the diagnosis was supported by characteristic histopathological findings and the isolation of dual species from tissue culture. Successful treatment was obtained following prolonged dual antibiotic therapy. This case highlights the importance of considering mixed NTM infection in chronic cutaneous eruptions and recognizing the potential pathogenic role of *M. septicum*.

## Conflicts of interest

None disclosed.
